# Multicentre, double-blind, randomised, sham-controlled trial of 10 khz high-frequency spinal cord stimulation for chronic neuropathic low back pain (MODULATE-LBP): a trial protocol

**DOI:** 10.1186/s13063-019-3831-4

**Published:** 2020-01-28

**Authors:** Adnan Al-Kaisy, Jonathan Royds, Stefano Palmisani, David Pang, Samuel Wesley, Rod S. Taylor, Andrew Cook, Sam Eldabe, Lance McCracken, Rui Duarte, Jeremy Fairbank

**Affiliations:** 1 Pain Management & Neuromodulation Centre, St Thomas’ Hospital, Westminster Bridge Road, SE1 7EH London, UK; 2 Guys & St. Thomas NHS Foundation Trust, London, UK; 3 Institute of Health and Well Being, University of Glasgow, Glasgow, UK; 4 Institute of Health Research, University of Exeter Medical School, Exeter, UK; 5 Wessex Institute, University of Southampton, Southampton, UK; 6 University Hospital Southampton NHS Foundation Trust, Southampton, UK; 7 South Tees Hospitals NHS Foundation Trust, Middlesbrough, UK; 8 Department of Psychology, Uppsala University, Uppsala, Sweden; 9 Liverpool Reviews and Implementation Group, Health Services Research, University of Liverpool, Liverpool, UK; 10 University of Oxford, Oxford, UK

**Keywords:** Spinal cord stimulation, Neuropathic pain, Chronic neuropathic low back pain, Neuromodulation

## Abstract

**Introduction:**

Chronic neuropathic low back pain (CNLBP) is a debilitating condition in which established medical treatments seldom alleviate symptoms. Evidence demonstrates that high-frequency 10 kHz spinal cord stimulation (SCS) reduces pain and improves health-related quality of life in patients with failed back surgery syndrome (FBSS), but evidence of this effect is limited in individuals with CNLBP who have not had surgery. The aim of this multicentre randomised trial is to assess the clinical and cost-effectiveness of 10 kHz SCS for this population.

**Methods:**

This is a multicentre, double-blind, randomised, sham-controlled trial with a parallel economic evaluation. A total of 96 patients with CNLBP who have not had spinal surgery will be implanted with an epidural lead and a sham lead outside the epidural space without a screening trial. Patients will be randomised 1:1 to 10 kHz SCS plus usual care (intervention group) or to sham 10 kHz SCS plus usual care (control group) after receiving the full implant. The SCS devices will be programmed identically using a cathodal cascade. Participants will use their handheld programmer to alter the intensity of the stimulation as per routine practice. The primary outcome will be a 7-day daily pain diary. Secondary outcomes include the Oswestry Disability Index, complications, EQ-5D-5 L, and health and social care costs. Outcomes will be assessed at baseline (pre-randomisation) and at 1 month, 3 months and 6 months after device activation. The primary analyses will compare primary and secondary outcomes between groups at 6 months, while adjusting for baseline outcome scores. Incremental cost per quality-adjusted life year (QALY) will be calculated at 6 months and over the lifetime of the patient.

**Discussion:**

The outcomes of this trial will inform clinical practice and healthcare policy on the role of high-frequency 10 kHz SCS for use in patients with CNLBP who have not had surgery.

**Trial registration:**

Clinicaltrials.gov, NCT03470766. Registered on 20 March 2018.

**Disclaimer:**

The views expressed here are those of the authors and not necessarily those of the NHS, the NIHR or the Department of Health. The NIHR had no role in the study design, writing of the manuscript or the decision to submit for publication.

**Roles and Responsibilities:**

AK, SP, DP, SW, RST, AC, SE, LM, RD and JF all contributed to the trial design and to securing trial funding. AK, JR, SP, DP, and SE are involved in the recruitment, the intervention and the follow-up. SW will perform data collection and analysis. RST will be responsible for the statistical analysis, and RD will be responsible for the health economic analysis. All authors read and approved the final manuscript.

## Introduction

The prevalence of chronic low back pain in adults worldwide is estimated to range from 12 to 28% [1–4]. This leads to prolonged disability and time lost from work for those affected [5]. Within this group, an estimated 12–15% suffer from chronic neuropathic lower back pain (CNLBP), have relatively greater pain severity and account for more of the costs of this condition [6, 7]. Neuropathic pain is defined as a lesion or disease of the somatosensory system. Commonly used therapies for low back pain are largely ineffective for CNLBP [8].

The National Institute for Health and Care Excellence (NICE) recommends spinal cord stimulation (SCS) for refractory neuropathic pain. SCS is routinely used for people with predominantly neuropathic radicular pain that typically results from, or persists after, spinal surgery (so-called failed back surgery syndrome [FBSS]) [9, 10]. SCS has demonstrated cost effectiveness for this indication [11]. However, because of a lack of existing evidence and the difficulty in obtaining paraesthesia over the lower back, SCS has not been commonly used for treating patients with back pain who have not had spinal surgery [10, 12].

High-frequency 10 kHz SCS (Nevro, Redwood, CA, USA) is a recent advance in SCS technology. The current is delivered at a frequency of 10 kHz, as opposed to the 40 to 60 Hz generated by conventional SCS [13]. The key advantage of a higher frequency current is its apparent superiority to conventional SCS in targeting residual low back pain following back surgery [14]. Moreover, it does not generate any stimulation-related sensations, known as ‘paraesthesias’ that can become intolerable [14, 15]. An advantage in this absence of paraesthesia is that 10 kHz SCS provides the opportunity for sham-controlled and double-blind studies in the field of SCS, without the need for device modifications.

Our group has conducted an uncontrolled, multicentre, single-arm study in which 72 patients with significant low back pain with or without leg pain were implanted with a 10 kHz SCS [16]. This was a mixed cohort of patients with and without prior spinal surgery. At 24 months, the mean reported visual analogue scale (VAS) score for back pain was 3.3 (SD 0.3) in 65 patients, compared with 8.4 (SD 0.1) at baseline (pre-implant) and 2.7 (SD 0.3) at 6 months [16]. A total of 60% of all patients were responders (> 50% reduction in back pain) at 24 months [16]. VAS is a psychometric response scale used to measure pain severity between 0 and 10 cm, with 10 cm indicating the worst imaginable pain experienced [17]. Similar improvements were observed in leg pain, disability and sleep, with marked reductions in medication intake [16].

In a more recent multicentre randomised controlled trial (RCT), 10 kHz SCS therapy demonstrated superiority to conventional tonic SCS in the treatment of FBSS. A total of 198 participants with both back and leg pain were randomised to 10 KHz SCS or conventional SCS. The 10 kHz SCS decreased the back pain intensity by 67% compared to 44% in the conventional SCS arm [18]. This decrease was sustained at 24 months [19].

The above-mentioned studies focused on neuropathic back pain in the context of patients with previous spinal surgery. However, a small subset of patients who had not received spine surgery and had received 10 kHz SCS therapy in these studies also experienced pain relief and functional improvements comparable to those of patients with FBSS [14, 18].

We hypothesised that patients with CNLBP who had no prior spine surgery would benefit from 10 kHz SCS. To evaluate this hypothesis, we initially designed and conducted an open-label, uncontrolled, pilot study in 21 patients with CNLBP and no prior spine surgery. The 10 kHz SCS therapy significantly reduced the VAS for back pain intensity by a mean of 5.59 (SD 1.80) at 12 months in patients with medically refractory low back and with no past history of spine surgery. Of the implanted patients, 90% were classified as responders (i.e. VAS back pain reduction > 50%) at 12 months. We also observed a significant increase in the physical function scores and health-related quality of life at one year post-10 kHz SCS implantation. The mean pain intensity was reduced by 73%, and the disability measured by the Oswestry Disability Index (ODI) was reduced by 48%. Opioid medication intake decreased by 64%, and the mean EQ-5D quality of life scores improved from 0.16 to 0.47. Remarkably, 75% of patients were able to return to employment [20]. This improvement was sustained through the 3-year follow-up [21].

To date 10 kHz SCS has not been formally tested against a sham therapy, which is needed to isolate the specific therapeutic effects from those induced by placebo [22]. Possibly, some of the benefits reported may be nonspecific treatment effects (enhanced by a surgical procedure) or result from reporting bias in either the patient or assessor [22]. We have therefore specifically designed this fully powered, double-blind, randomised, sham-controlled trial of 10 kHz SCS to address this major methodological limitation of previous studies.

### Objectives

Hypothesis: The addition of 10 kHz SCS to usual medical care (intervention group) will provide superior back pain relief, compared to sham stimulation plus usual medical care (control group) for CNLBP.

Aim: The overarching aim of this study is to demonstrate the efficacy, safety and cost-effectiveness of 10 kHz SCS in the treatment of CNLBP with no prior surgery.

### Trial Design

**Fig. 1:**
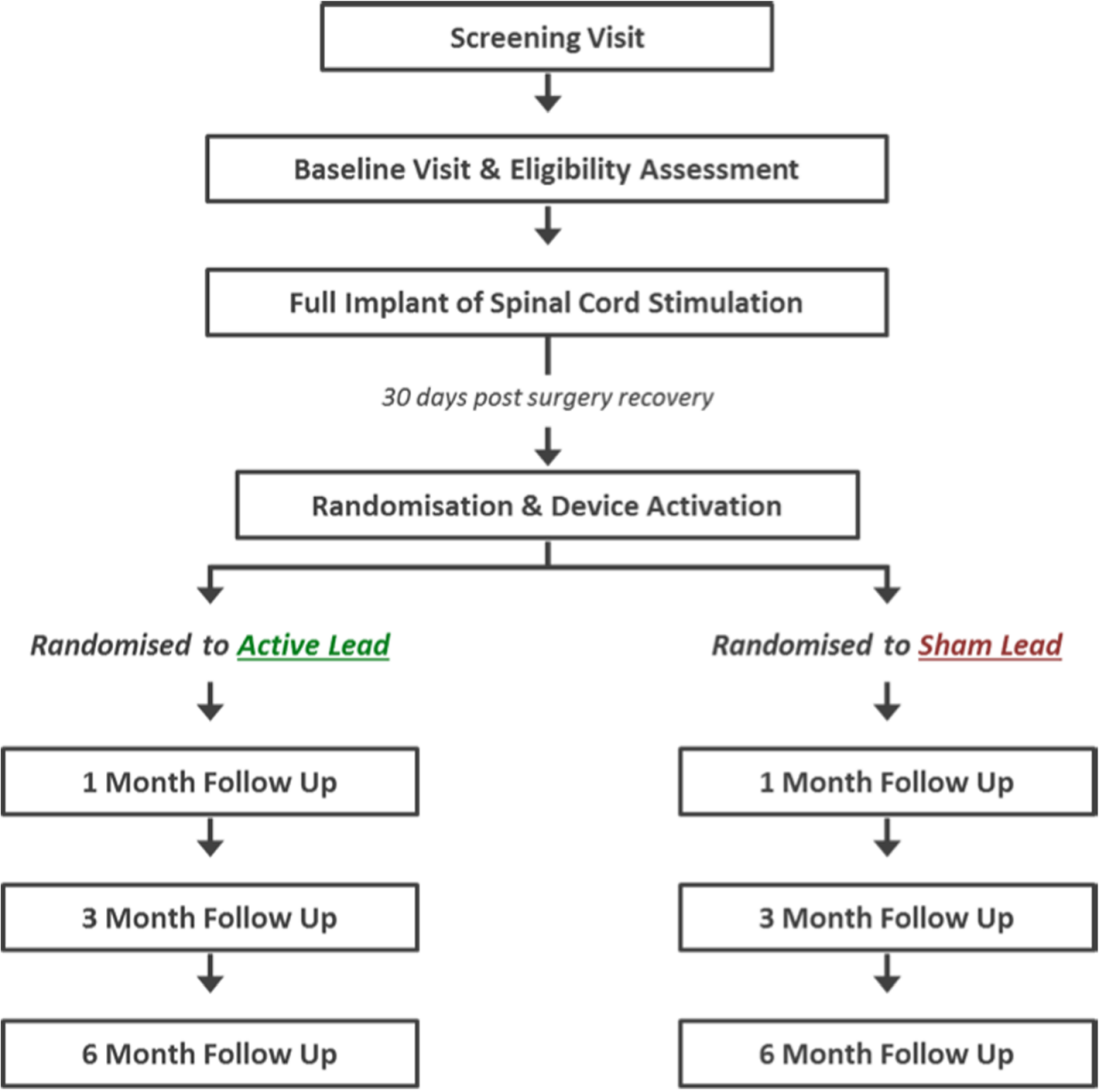
Consort diagram of MODULATE-LBP trial

This is a multicentre, randomised, double-blind, superiority, sham-controlled trial with parallel economic evaluation. Patients will be individually allocated to activated 10 kHz SCS plus usual care (intervention) or sham 10 kHz SCS plus usual care (control) and followed up for 6 months. A summary of the study CONSORT diagram is illustrated in Fig. 1.

## Methods

This protocol has been prepared and reported in accordance with the Defining Standard Protocol Items for Clinical Trials (SPIRIT) statement (Additional file 2) [23].

### Study Setting

Participants will be recruited from two neuromodulation centres: Guy’s & St. Thomas NHS Foundation Trust, London, UK, and South Tees Hospitals NHS Foundation Trust, Middlesbrough, UK.

### Patient and Public Involvement

We organised three Patient and Public Involvement and Engagement (PPIE) meetings to discuss research design elements that would be acceptable to patients for a condition that has been resistant to conventional medical management. On 8 April 2016 a PPIE event was held at the biannual scientific meeting of the UK Spine Societies (BritSpine). At this event 7 patients and 22 professionals attended a session to provide feedback and advice on this application. On 24 July 2015 Guy’s & St Thomas’ Hospital hosted a PPIE meeting of participants from our pilot study [16]. Eleven patients and their relatives attended. In addition, another 10 patients attended a subsequent meeting on 11 November 2016. The outline of the proposed study was presented during both meetings, where the patients were asked to enrol in the study and to give their general thoughts on the study methods. Two experienced facilitators from the local Research and Design Service (RDS) fielded questions on recruitment, comparison treatments, blinding, outcome measures and methods and the dissemination of results. Feedback was collected via assessment sheets, reviewed and incorporated into the study design. Patient and public engagement meetings occur annually through the duration of the study, with an additional dissemination meeting scheduled for the final year.

During the PPIE Meetings a number of points were raised:

1. The overall response to these meetings has been thoroughly positive, and patients have expressed their enthusiasm for this trial and willingness to attend future meetings.
2. Patients described long-standing chronic pain, inadequate treatments and the need for long-lasting therapy without medication.
3. Patients expressed a willingness to join the trial and be allocated 50/50 to an active or inactive treatment, with assurances being provided about device activation at the end of the study.
4. Patients accepted the inclusion of a sham arm, but we abandoned our original plan to cross over at 6 months and extend the trial to 12 months. We felt that this approach would be unfair to patients who were allocated active therapy that was then withdrawn.
5. A 6-month blinded period was accepted as a reasonable balance between patient and research needs, but 12 months was too long.
6. Patients emphasized measures related to their broader experiences, such as physical function, disability and goal-orientated outcomes rather than just pain.
7. Patient travel reimbursement maximums were raised from £30 per visit to £50 per visit, as £30 was deemed insufficient for patients travelling from outside of London.
8. Throughout these meetings, the patients have an appointed PPIE representative, Mr. Dean Walker. Dean was a participant in our pilot study, joined the research team before this application was first drafted and agreed to be a co-applicant. He has attended all our PPIE events, has participated fully as a member of the applicant team and will remain on the research team until completion of the study.

### Eligibility Criteria

The intended study population includes individuals with CNLBP who have not had surgery. Participants will be assessed for eligibility using the study-specific inclusion/exclusion criteria during the screening visit.

### Inclusion Criteria

Individuals are included according to the following criteria:

1. 18-years old at the time of consent
2. Willing and able to sign and date the informed consent form
3. Capable of independently comprehending and consenting to the requirements of the study
4. Willing and able to comply with all study procedures and study visits and available for the duration of the study
5. Diagnosed with low back pain with VAS pain scores ≥60 out of 100 mm for at least 12 consecutive months
6. Low back pain of greater intensity than any leg pain
7. Presence of clear component of neuropathic pain based on a painDETECT Questionnaire score of ≥19 [24]
8. Degenerative disc disease confirmed by imaging or internal disc degeneration as confirmed by discography
9. Stable dose (no new, discontinuation of or change in) of all prescribed pain medications for at least 4 weeks prior to screening and willing to maintain or only decrease the dose of all prescribed pain medications through Trial Assessment 2.
10. Has tried appropriate conventional medical management for the pain

### Exclusion Criteria

Individuals are excluded according to the following criteria:

1. Presence of an active neurostimulator implanted device, whether turned on or off
2. Previous spinal surgery
3. Current signs of a systemic infection
4. Pregnant or lactating, inadequate birth control, or the possibility of pregnancy during the study
5. Current diagnosis of a progressive neurological disease such as multiple sclerosis, chronic inflammatory demyelinating polyneuropathy, rapidly progressive arachnoiditis, rapidly progressive diabetic peripheral neuropathy, brain or spinal cord tumour, or severe/critical central or foraminal spinal stenosis
6. Mechanical spine instability detected by a clinician (validation by flexion/extension films of lumbar spine in the past 12 months showing 4 mm or more translational movement or excessive angular movement manifested by > 5 degrees segmental angular movement), e.g. any forms of spondylolisthesis
7. A medical condition or pain in other area(s) that is not the condition intended for treatment with SCS and could interfere with the study procedures, accurate pain reporting and/or confound evaluation of the study endpoints, as determined by the Investigator
8. Evidence of an active disruptive psychological or psychiatric disorder or other known condition significant enough to impact the perception of pain, compliance of intervention and/or ability to evaluate treatment outcome as determined by the Investigator
9. Significant drug-related behavioural issues (e.g. alcohol dependency, illegal substance abuse)
10. Using greater than 120 mg morphine equivalents of opioids daily
11. Structural abnormalities of the spine that may prevent electrode implantation
12. Co-existing disorder of the nervous system that may affect study measurements, e.g. polyneuropathy
13. Diagnosed with fibromyalgia or other generalised pain syndromes
14. Active malignancy or diagnosis of cancer and not in remission for at least 1 year prior to screening
15. Participating or planning to participate in another clinical trial
16. Patient is in close contact with other people involved with the study so that a high risk exists for the patient to be unblinded.

### Interventions

#### Removal of ‘Temporary Trial’ Phase from Design

A trial of stimulation is often performed in standard care to eliminate non-responders prior to full implantation This temporary trial of stimulation is performed by implanting the leads and connecting them via a temporary extension to an external battery that the patient can wear for up to 2 weeks. This is a recommendation of the current NICE guidelines and the manufacturer’s device manual.

RCT evidence does not support the role of temporary screening trials for stimulation in predicting long-term outcomes of the therapy [25]. Positive evidence exists of harm from temporary screening trails, including higher infection rates and the potential elimination of long-term responders [26]. In our preliminary study on 10 kHz stimulation for patients with low back pain without prior surgery, we noted a trial success rate of 95% [20]. This high success rate was credited to more stringent patient selection, based on the characterisation of pain mechanisms and other clearly defined inclusion criteria. By removing the trial phase we reduce the number of procedures that patients undergo from two to one.

Therefore, we assert that a pre-implantation trial of 10 kHz SCS in this population offers little clinical value and, instead, increases the costs and harms; in the context of the present trial, it would reduce the scientific value of this research.

#### Identification and Description of the Investigational Device

The Senza™ System, a totally implantable SCS system that is intended to aid in the management of chronic intractable pain, received a European Union (EU) CE Mark in May 2010.

The Senza System consists of a rechargeable implantable pulse generator (IPG) with 16 output channels. The IPG is implanted in a subcutaneous pocket and is capable of stimulating the spinal cord nerves when used with one or two 8-contact percutaneous leads. The IPG is controlled by a patient remote and/or a clinician programmer.

Lead(s): The percutaneous lead has eight contacts.

Extension(s): An extension may be used during the permanent implantation procedure, to connect the lead to the IPG.

IPG: The IPG is a rechargeable stimulator with 16 output terminals. Each of the 16 outputs can be programmed as a cathode or an anode. The IPG is powered by a 3.6 V nominal lithium-ion rechargeable battery. It is capable of stimulating the spinal cord nerves through the electrodes of the leads connected to any combination of the output terminals, using one programmable current source.

Patient remote control: The patient remote control is a handheld battery operated unit that the participant can use to turn the stimulation on and off and to change the stimulation programs/settings.

Charger: This charger is used to transcutaneously charge the IPG battery. It is a portable device powered by a rechargeable battery and can be held in one hand.

Clinician programmer: The clinician programmer is an off-the-shelf laptop installed with proprietary software to allow the programming of the IPG and participant remote control.

Lead anchors: Lead anchors may be used to secure the leads to the fascia, possibly preventing lead migration and/or lead strain.

**Fig. 2:**
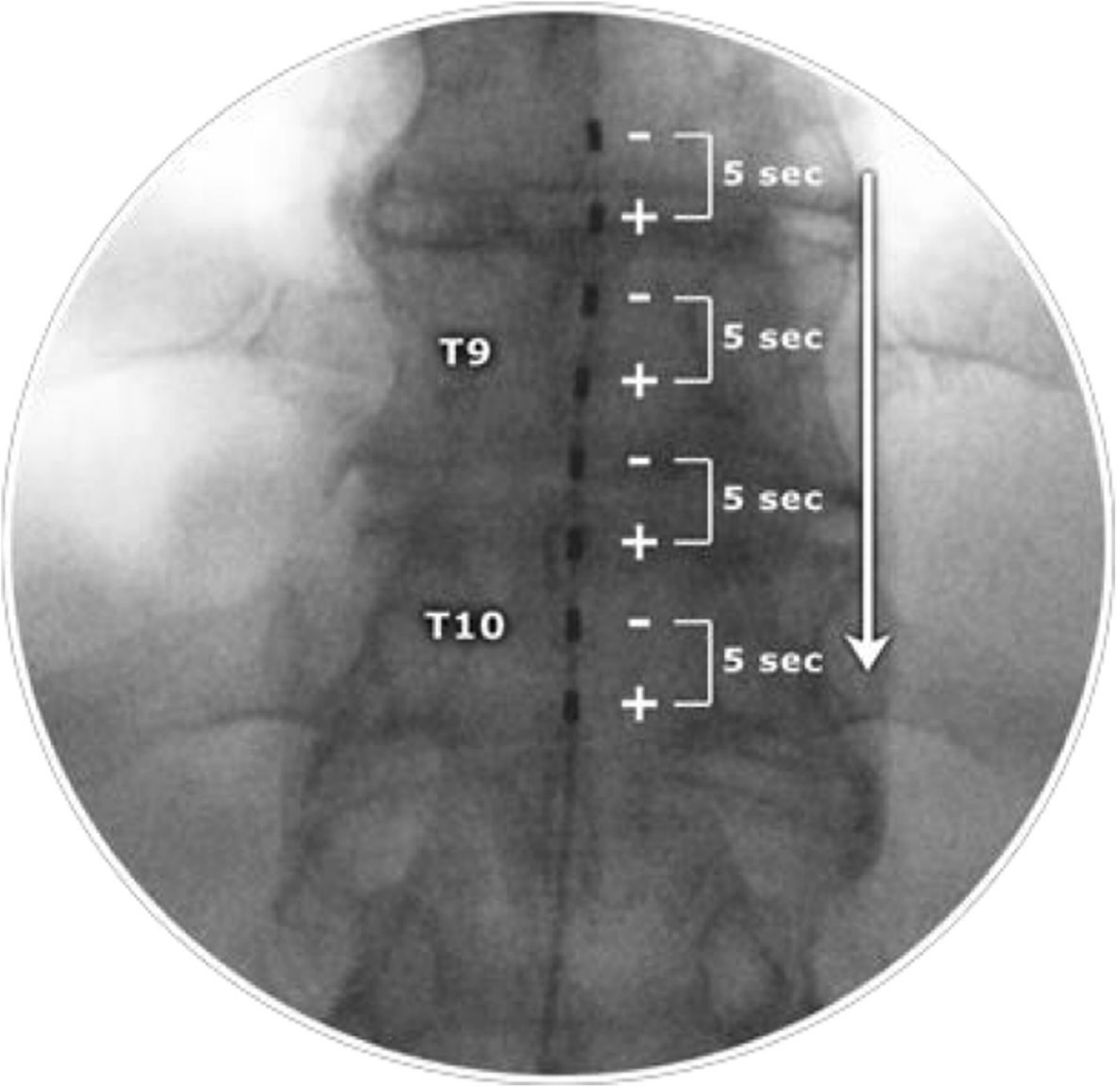
Anterior-posterior X-ray position of desired lead location and diagrammatic illustration of ‘cascade’

We will be using cascade programming in both groups; this involves four pairs of electrode groups and each pair is switched on for 5 s before the next pair is switched on (Fig. 2). Therefore, in 20 s, the whole lead has been activated and the cycle repeats. The rationale is that this avoids overstimulation, mitigates against small degrees of lead migration and removes any need for reprogramming. During the study, as both groups are programmed identically and reprogramming is not expected, the risk is reduced for unblinding and subsequent bias.

### Device Implantation

Under standard operating theatre practice, all participants will be implanted with the following equipment: 10 kHz Senza IPG and two octopolar (8 contact) leads.

The first of the two leads will enter the epidural space through a lumbar or lower thoracic epidural puncture. The lead will be advanced cranially in the epidural space to reach a final position, where contacts 4 and 5 span the T9/10 disc space shown by Anterior-Posterior fluoroscopy in the anatomical midline as per our pilot experiment (Fig. 2). A lateral image will be obtained to ensure the lead is placed posteriorly in the epidural space.

**Fig. 3:**
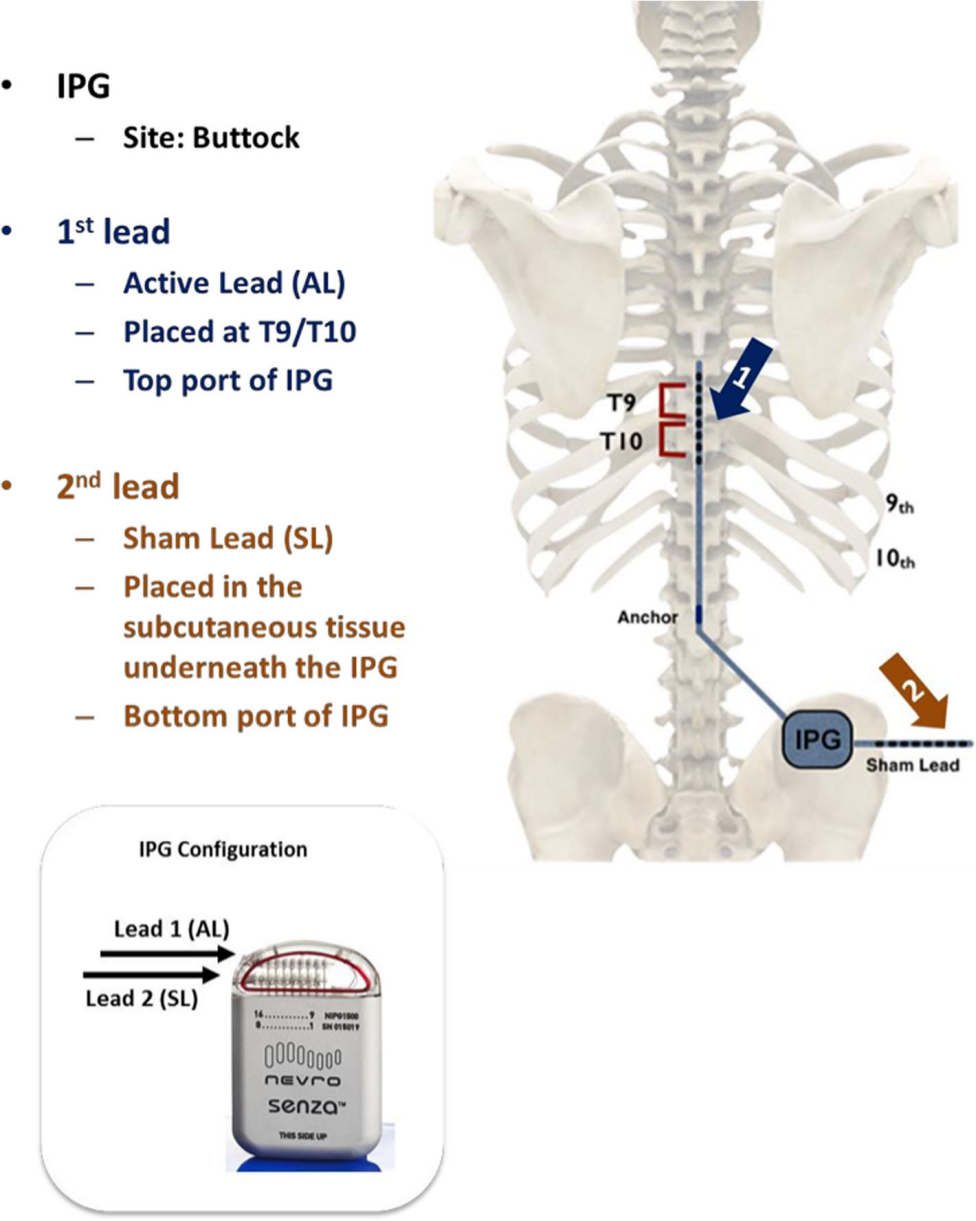
Position of epidural lead (1), sham lead (2) and implantable Pulse Generator

Once satisfactory epidural lead placement is confirmed, the lead will be anchored to the deep fascia or supraspinous ligament and a strain relief loop will be established. The active epidural lead will be tunnelled to a subcutaneous pocket for the battery using an extension if necessary. This subcutaneous pocket is made in the gluteal region via a small skin incision and blunt subcutaneous dissection. This pocket should be large enough to accommodate the IPG, extensions and sham lead. This active lead (AL) will be used to provide therapy to the intervention group. The AL will be connected to the first or top port of the IPG in all participants, and a second sham lead (SL) will be inserted subcutaneously and attached to the second port of the IPG (Fig. 3). Impedances to check electrical integrity of the system will be performed at this time. Information will be given about wound care, and the device will remain ‘off’ until the participant’s next visit. Removal of sutures (if necessary) will be done at the implantation site.

Possibly, the SCS will need removal or repair. Reasons for these actions include patient withdrawal from study, infection, hardware failure, lead migration, SCS-related pain, allergy or other adverse reaction to the device or requirement for an MRI scan.

If this occurs, the participant must consent to a further surgical procedure, which is a surgical process similar to that of implantation. With removal, all hardware will be removed, and with repair, the changes made to the SCS components will be determined by which component is unsatisfactory. The risk from these surgeries is similar to that of implantation, with an additional small risk that some of the components may not be safely removed.

All such procedures will be done by the study implanter.

### Outcomes

#### Primary Outcome

The primary outcome is the comparison between the intervention and the control of the effects of stimulation delivered on the mean VAS back pain scores taken from the 7 days of diary data from the week prior to and from the 6 months following post-randomisation.

#### Secondary Outcomes

The secondary outcomes include the following:

- A comparison of the secondary outcomes of disability, depression, health-related quality of life, patient’s global impression of change, sensation maps and medication usage between intervention and control at 1, 3, and 6 months post-randomisation
- A comparison of the cost-effectiveness of 10 kHz SCS between intervention and control at 6 months post-randomisation
- A comparison between the intervention and control of the complications and adverse events at 6 months post-randomisation

### Participant Timeline

#### Screening Visit

**Fig. 4:**
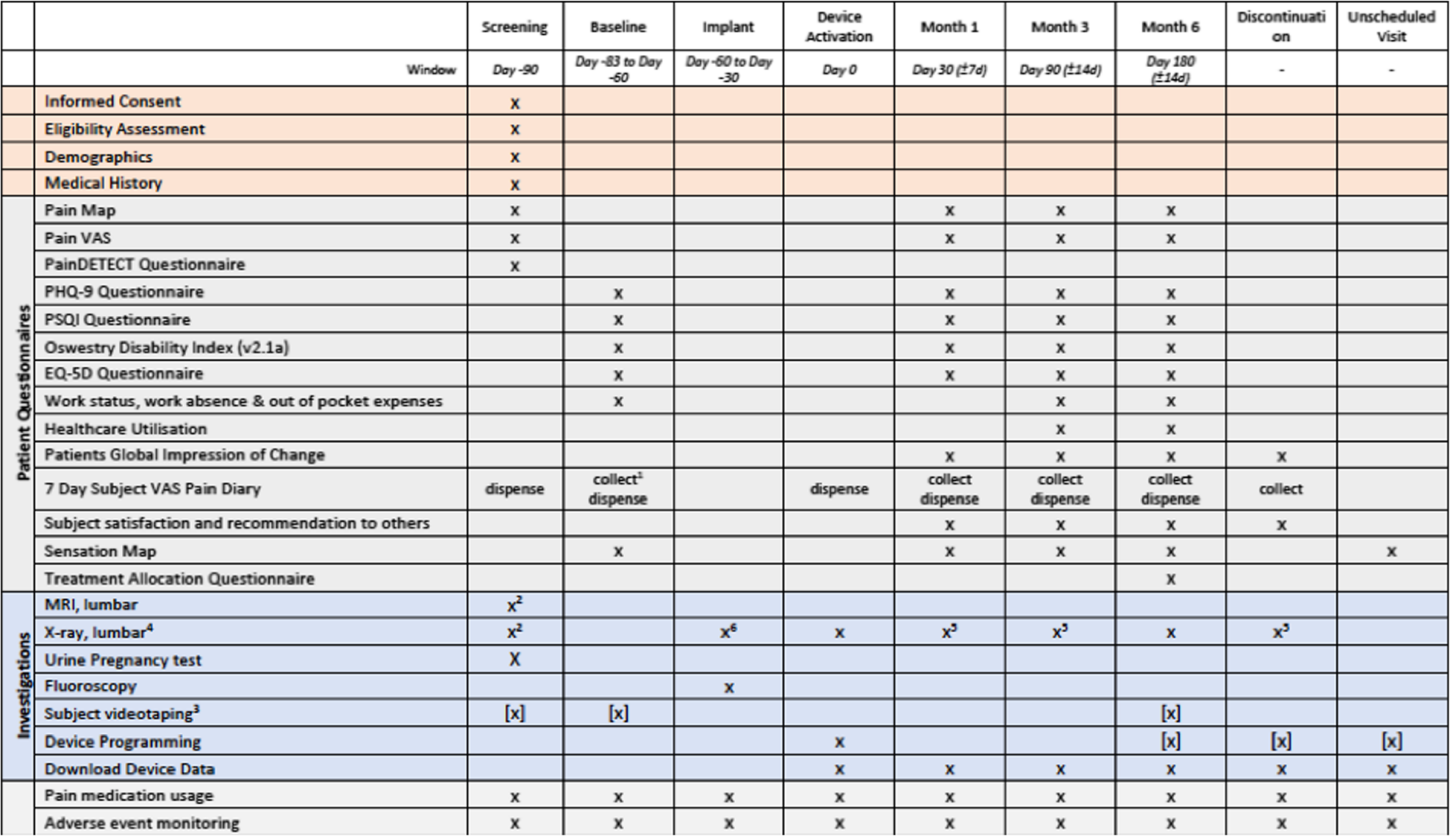
Standard Protocol Items: Recommendations for Interventional Trials (SPIRIT) diagram

The schedule of events is listed in Fig. 4. Participants will be given a copy of the patient information sheet and informed consent form (Additional file 1); they will be provided sufficient time to read and understand the document and the opportunity to ask questions. Participants will be informed of their right to withdraw from the study at any time without prejudice.

The screening visit will commence after each participant has been enrolled. During the screening visit, the following will be collected: eligibility assessment, participant demographics, medical history, pain map, pain severity on a 0-100 mm (VAS), painDETECT questionnaire, pregnancy test from all female participants of child-bearing potential, and pain medication usage.

If a participant reports a painDETECT score lower than 19, they will be discontinued from the study. During the screening visit, participants will be provided with a multi-day diary. Participants will record their VAS back and leg pain scores for 7 days prior to the baseline visit.

#### Baseline Visit

During the Baseline visit, participants will be asked to complete the following questionnaires after being provided detailed instructions: the Oswestry Disability Index (ODI v2.1a), Patient Health Questionnaire (PHQ-9), Pittsburgh Sleep Quality Index (PSQI), EQ-5D-5 L, pain medication usage, work status and work absence. If a participant reports a mean VAS back pain score lower than 60 mm on their multi-day pain diary, they should be discontinued from the study.

### Randomisation and Device Activation

The randomisation and device activation visit will take place between 2 and 4 weeks post-implantation, pending proper wound healing.

Firstly, the following will be performed by blinded study personnel:

- Participants will be assessed to determine whether they have experienced any adverse events, and a case report form (CRF) will be completed, as applicable.
- New x-ray images will be taken to record the lead locations.
- Participants will be provided with a multi-day diary and instruct to record their VAS pain scores daily for 7 days prior to the 1-month follow-up.
- The participant’s device and lead will be programmed according to their allocated group; whereas the lead is different, the programming parameters used are identical and can be found in Fig. 2.

At this point, the participant enters the ‘follow-up’ phase of the study, with visits at 1, 3 and 6 months. These visit frequencies mirror our standard of care for routine clinical practice.

During these visits, all participants will be asked to complete the following questionnaires in addition to bringing their multi-day pain diary:

- Pain severity on a 0-100 mm (VAS)
- Pain map
- Oswestry Disability Index (ODI v2.1a)
- Patient Health Questionnaire 9 (PHQ-9)
- Pittsburgh Sleep Quality Index (PSQI)
- EQ.5D-5 L
- Pain medication usage
- Patient Global Impression of Change (PGIC)
- Participant satisfaction
- Sensation map.

Any adverse events are monitored and documented in the CRF. An X-ray may be taken if there are any concerns about lead migration.

To check fidelity of blinding, participants and assessors will be asked to guess their treatment allocation at the end of the study. We will monitor potential outcome assessor blinding by asking them to guess each patient’s allocation at the end of each follow-up assessment. After the trial is completed, we will compare actual and anticipated treatment allocations.

### After 6-month Follow-up

After the 6-month visit all patients will be managed in the standard pain management service, outside the trial.

### Medication Usage

All medications prescribed for the treatment of pain will be recorded during the screening visit. Only participants who are on a stable dose of all prescribed pain medications for at least 4 weeks prior to screening and willing to maintain or decrease the dose of all prescribed pain medications are eligible for participation in this study. Any changes to an enrolled participant’s pain medications during the study will be recorded.

The addition of pain medications for the relief of surgical discomfort after implant procedures is allowed. Pain medications prescribed for short-term post-operative pain management are not considered an increase in pain medications if the medication is ceased and the medication end date is prior to the date of randomisation.

All medications used to treat adverse events (regardless of the reason prescribed) will be documented as interventions on the Event CRF.

### Sample Size

For our primary outcome of pain severity VAS (0-100 mm), the Initiative on Methods, Measurement and Pain Assessment in Clinical Trials (IMMPACT) proposes a minimally important clinical difference (MICD) of 20 mm [27]. To detect this MICD and based on a back/leg pain VAS standard deviation of 25 mm, as seen in previous conventional and 10Khz-SCS RCTs set at 90% power and 5% alpha, and a worst case attrition rate of 30% at 6-months follow up, we will need to recruit 96 participants (48 per centre) per group.

### Recruitment

Recruitment will be from outpatients of the two centres. The study will be presented in national and international meetings to a target audience of pain physicians, spinal surgeons, neurosurgeons and physiotherapists. Advertising will be conducted through newspapers and magazines to enhance recruitment. Road shows were organised to target spine surgeons, neurosurgeons and pain physicians.

### Assignment of Intervention

#### Allocation: Sequence Generation

Patients will be randomised using a validated and password-protected trial website, designed and supported by UK CRC registered Exeter Clinical trials unit (CTU).

#### Allocation Concealment

Patients will be allocated 1:1 to intervention or control and were stratified by centre using a web-based computer-generated random-allocation sequence to ensure concealment. This website will be password protected and managed by Exeter CTU.

#### Blinding

Patients, investigators, outcome assessors and analysts will all be blinded. Only two study nurses at each site will be unblinded to the treatment received by the participants.

#### Data Collection/Management

Data analyses will be conducted and reported in accord with CONSORT (Consolidated Standards Of Reporting Trials) guidelines for non-drug trials [28]. Participant flow will be summarised using a flow diagram, which will report recruitment and drop-outs and baseline patient characteristics and outcome scores reported and compared by group. The primary analysis for the primary and secondary outcomes will take an intention-to-treat approach based on a between-group comparison of intervention and control participants with complete data at 6 months, with adjustments being made for the baseline outcome score and centre. In a secondary analysis we will extend the primary analysis model with a repeated measures comparison of groups at all follow-up points. We comprehensively examine patterns and reasons for the missing outcome data and undertake appropriate imputation models to assess the impact of missingness on primary analysis models. Results will be reported for between-group mean differences and 95% confidence intervals. Safety outcomes will be reported descriptively by group. No correction of *P* values for multiplicity of testing will be undertaken. However, the primary outcome analysis will be performed before all other analyses, and the *P* values of all subsequent analyses will be interpreted in the context of multiple testing. No interim analyses are planned.

### Economic Evaluation Methods

The economic evaluation aims to estimate the cost-effectiveness of 10 kHz SCS plus usual medical care when compared to sham stimulation plus usual medical care. Healthcare resource utilisation, e.g. management of adverse events, interventions, investigations, medication, inpatient hospitalisations, Emergency Department and other health-care related visits, plus out-of-pocket costs and absences from work will be collected for each patient during the study follow-up period. Resources required for the implantation intervention will be recorded within the trial.

Items of resource use will be costed using national averages obtained from national sources (such as the Personal Social Services Research Unit, the British National Formulary and National Health Service (NHS) reference cost databases). Cost components will be combined to derive total patient level costs for the NHS. In addition, non-NHS costs such as productivity loss due to absence from work or patient out-of-pocket expenses will also be quantified to provide a full picture of how the strategies being compared will affect the financial burden imposed by the condition on both the NHS and the patients.

Generic health-related quality-of-life (HRQoL) data will be collected using the EQ-5D-5 L instrument. Both resource utilisation (costs) and EQ-5D-5 L will be collected at each follow-up visit. A within-trial cost consequence analysis will be carried out to estimate mean resource utilisation, costs, EQ-5D scores and total quality-adjusted life years (QALYs) in each group, together with relevant measures of sampling uncertainty. QALYs will be calculated using the area under the curve approach, with regression-based adjustment for the baseline EQ-5D score.

The economic evaluation will take the form of a cost-utility analysis to calculate the cost per additional QALY gained. Base-case analyses will be conducted from the NHS perspective, with additional analyses being conducted from the societal perspective. Deterministic and probabilistic sensitivity analysis will be undertaken to explore the robustness of the results to plausible variations in key assumptions and variations in the analytical methods used. Cost-effectiveness acceptability curves will be constructed to show the probability that the intervention is cost-effective at specific thresholds of cost per QALY gained. The analyst will be blinded to the group allocation.

### Statistical Methods

The trial statistician will be blinded to the group allocation and will undertake analyses using STATA. A detailed statistical analysis plan (SAP) will be prepared that will be presented to the Trial Steering Committee (TSC) and Data Monitoring Committee (DMC) for their review ahead of any analysis being undertaken.

### Data Monitoring

A joint Trial Steering Committee (TSC) and Data Monitoring Committee (DMC) will provide supervision for the trial, providing advice to the Chief and Co-investigators (Trial Management group [TMG]) on all aspects of the trial conduct and affording protection for patients by ensuring the trial is conducted according to the Medical Research Council (MRC) Guidelines for Good Clinical Practice in Clinical Trials. The TSC/DMC will be chaired by an academic clinician independent of the trial plus three other members plus the chief investigator, trial manager, statistician and health economist.

### Harms

#### Foreseeable Adverse Events and Anticipated Adverse Device Effects

We expect the risk of serious adverse events to be rare, and our results from the previous 10 kHz stimulation trial showed the risk of adverse events to be as follows:

- Battery pocket pain 8%
- Wound infection 3%
- Electrode migration 5%
- Lead Fracture or malfunction 6%
- Headache from epidural puncture 1%
- Nerve injury < 1%
- Spinal cord haematoma and abscess < 1%
- Unwanted, perceived stimulation < 1%

Pocket pain is usually self-limiting within 6 months, and electrode migration is mitigated using cascade stimulation programming. Wound infection will necessitate the removal of the device. In some instances the system will require repair or removal. The reasons for this include infection, hardware displacement, pain or discomfort from the device, or device failure. The risks are similar to those mentioned above.

All adverse events will be recorded and reported in accordance with Good Clinical Practice.

#### Device Repair or Removal

Possibly, the SCS will need to be removed or repaired. Reasons include patient withdrawal from study, infection, hardware failure, lead migration, SCS related pain, allergy or other adverse reaction to device, or a required MRI scan.

If this occurs, the participant must consent to a further surgical procedure, and the surgical process is similar to that of implantation. With removal, all hardware will be removed, and with repair, the changes necessary to the SCS components will be determined by which component is unsatisfactory. The risk from these surgeries is similar to that of implantation, with a small risk that some of the components may not be safely removed. All such procedures will be done by the study implanter. Any adverse events are monitored and documented in the CRF.

### Auditing

The TSC will meet three times in the first year, twice in the second year and three times during the third year. The DMC will meet twice in the first year and annually thereafter.

If the chief and co-investigators are unable to resolve any concern satisfactorily, personal investigators, and all others associated with the study, may write to the chairperson of the TSC, through the trial office, drawing attention to any concerns they may have about the possibility of particular side-effects or about any other matters thought relevant. Interim analyses of recruitment rate, safety and outcome data will be supplied, in strict confidence, to the committee, along with any other analyses that the committee may request.

### Ethics and Dissemination

#### Declaration of Helsinki, International Standards and National Regulations

The clinical investigation shall be conducted in accordance with the ethical principles of the Declaration of Helsinki, ISO 14155, and all other applicable device and UK regulations.

Central ethical approval was provided by London-Camberwell St. Giles Research Ethics Committee for both centres, part of the NHS Health Research Authority. REC reference: 18/LO/1031, IRAS project ID: 232729. We will not begin recruiting at other centres in the trial until local ethical approval has been obtained. Informed written consent will be obtained prior to trial participation.

#### Protocol Amendments

Any protocol amendments will be discussed and approved within the TMG and presented to inform the TSC/DMC.

### Consent

The principal investigator or qualified designee will document the informed consent process, including the date of consent and name of the person conducting the consent process in the participant’s medical record. Individuals will be considered enrolled in the study once they have signed the informed consent form (Additional file 1). On the consent form, participants will be asked if they agree to the use of their data by responsible and designated individuals in the Foundation trust and regulatory authorities, the Exeter CTU and other collaborators. Participants will also be asked for permission to be contacted about future research projects, where relevant. This trial does not involve collecting biological specimens for storage.

### Confidentiality

All data will be handled in accordance with the UK Data Protection Act 1998. The CRFs will not bear the participant’s name or other personal identifiable data. The participant’s initials, date of birth and trial identification number will be used for identification. Participants will be assigned a trial identification number by the study site sequentially upon enrolment into the study. The study site will maintain a master participant identification log.

### Dissemination

The results of this trial will be reported and presented at national and international meetings. The outcomes will be published in high-quality, peer-reviewed journals to make the results available to other physicians and scientists. Further long-term outcomes will be reported to potentially validate the longevity of the therapy.

## Discussion

The results of this study will demonstrate whether the application of 10Khz-SCS in patients with CNLBP is a suitable alternative to conventional medical therapy. Multiple large-scale trials involving the use of SCS thus far have been industry-sponsored without the use of a placebo group. Many studies have chosen not to use a placebo arm with a high probability of unblinding due to the nature of SCS. The methods used in this trial attempt to ensure that unblinding does not take place. The sham lead positioned outside the epidural space ensures energy consumption without neurostimulation, requiring the patient to recharge the device. None of these participants will have had exposure to SCS prior to the trial, so the experience of the therapy will be novel. As this is the first trial to apply these concepts, the results will act as a support for future trials evaluating the efficacy of SCS in other pathologies.

### Trial Status

The current protocol version is V1.2 dated 18 December 2018. The start date of the study was 14 August 2018, and patients are currently being recruited. Recruitment is expected to be completed by 1 December 2020. The estimated primary completion date is 1 July 2021.

## Supplementary information

**Additional file 1:** Consent form.

**Additional file 2:** SPIRIT 2013 Checklist: Recommended items to address in a clinical trial protocol and related documents*.

## Data Availability

The datasets generated and/or analysed during the study are available from the corresponding author on reasonable request.

## Abbreviations

AL: Active lead
CE: Conformité Européene
CNLBP: Chronic neuropathic low back pain
CONSORT: Consolidated Standards of Reporting Trials
CRF: Case report form
CTU: Clinical trials unit
DMC: Data monitoring committee
EQ-5D: European Quality of Life Score
EU: European Union
FBSS: Failed back surgery syndrome
IPG: Implantable pulse generator
LBP: Low back pain
MICD: Minimally important clinical difference
MRC: Medical Research Council
MRI: Magnetic resonance imaging
NHS: National Health Service
NICE: National Institute of Clinical Excellence
ODI: Oswestry Disability Index
PGIC: Patients Global Impression of Change
PHQ: Patient health questionnaire
PPIE: Patient and public involvement and engagement
PSQI: Pittsburgh Sleep Quality Index
QALY: Quality-adjusted life years
RCT: Randomised controlled trial
RDS: Research and design service
SAP: Statistical analysis plan
SCS: Spinal cord stimulation
SD: Standard deviation
SL: Sham lead
TMG: Trial management group
TSC: Trial steering committee
VAS: Visual analogue score
