# Supplementary material for: Multicentre, double-blind, randomised, sham-controlled trial of 10 khz high-frequency spinal cord stimulation for chronic neuropathic low back pain (MODULATE-LBP): a trial protocol
Source: Trials. 2020 Jan 28;21:111. doi: 10.1186/s13063-019-3831-4 (PMC6986091; doi:10.1186/s13063-019-3831-4)
Supplement: Supplementary file 1 — Additional file 1: Consent form. [file 13063_2019_3831_MOESM1_ESM.pdf]

Guy's and St Thomas' NHS
NHS Foundation Trust

Pain Management & Neuromodulation Centre
Ground Floor, Lambeth Wing
St Thomas' Hospital
Westminster Bridge Road
London SE1 7EH

Department: 020 7188 4716
Fax: 020 7188 4719
Main Switchboard: 020 7188 7188

INFORMED CONSENT FORM

Sponsor: Guy's and St Thomas' NHS Foundation Trust
Protocol number: Modulate-LBP v1.2 18th December 2018
Ethics Committee reference number: 18/LO/1031

Investigator: Dr Adnan Al-Kaisy
IRAS ID: 232729

**Study Title:**	**Multicentre, Double Blind, Randomised Sham-Controlled Trial of 10 kHz High-Frequency Spinal Cord Stimulation for Chronic Neuropathic Low Back Pain**		
**Protocol Number:**	**V1.2 18.DEC.2018**		
**Subject Number:**	01 ___	**Date of Birth:**	______ / ______ / _______

	Patient Initials
I confirm that I have read and understand the information in the patient information sheet (Version 1.9 dated 18th of December 2018) for the above named study. I have had sufficient time, the opportunity to consider the information, ask questions and have had these questions answered satisfactorily.	
I understand that my participation is voluntary and that I am free to withdraw at any time, without giving any reason, and without my medical care, treatment or legal rights being affected.	
I understand that relevant sections of my medical notes and data collected during the study may be looked at by responsible and designated individuals from Guy's and St. Thomas NHS Foundation Trust or regulatory authorities, where it is relevant to my participation in this study. I give permission for these individuals to have access to my records.	
I understand all information collected about me will be kept confidential	

INFORMED CONSENT FORM

Sponsor: Guy's and St Thomas' NHS Foundation Trust
Protocol number: Modulate-LBP v1.2 18th December 2018
Ethics Committee reference number: 18/LO/1031

Investigator: Dr Adnan Al-Kaisy
IRAS ID: 232729

	Patient Initials
I agree to my GP being informed of my participation in the study	
I consent to being contacted about future research projects	
I consent that my data being shared with Exeter clinical trial unit and other collaborators some of which may be outside of the UK	
I freely and voluntarily consent to participate in the study	
I understand that I will receive a signed copy of this consent and authorisation form. I also understand that a copy of the consent form will be filed with my medical records and a copy with the research team.	

Three copies of this consent form must be signed and dated by you and the investigator:

Site File Copy

**Name of Participant:**	**Signature:**	**Date:**
**Name of Person Obtaining Informed Consent:**	**Signature:**	**Date:**

INFORMED CONSENT FORM

Sponsor: Guy's and St Thomas' NHS Foundation Trust
Protocol number: Modulate-LBP v1.2 18th December 2018
Ethics Committee reference number: 18/LO/1031

Investigator: Dr Adnan Al-Kaisy
IRAS ID: 232729

Study Overview: **Multicentre, Double Blind, Randomised Sham-Controlled Trial of 10kHz High- Frequency Spinal Cord Stimulation for Chronic Neuropathic Low Back Pain (MODULATE-LBP)**

Patient ID:

Informed Consent Process

**1.** The subject was given adequate information concerning the study	O Yes O No
**2.** The subject was provided adequate opportunity to consider all options	O Yes O No
**3.** All of the subject's questions were responded to	O Yes O No
**4.** The subject comprehended the information given to him	O Yes O No
**5.** The subject personally signed and dated	O Yes O No
**6.** The subject received a signed copy of the informed consent form and patient information sheet	O Yes O No
**7.** The informed consent process occurred prior to study related activities beginning	O Yes O No
**8.** The subject is eligible according to the Inclusion/Exclusion criteria of the study	O Yes O No

I (study coordinator) witnessed the patient has been informed to these guidelines

Sign: ____

Print: ____

Date: _ _ / _ _ _ / _ _ _ _
